# Artificial Intelligence-Based Multiclass Classification of Benign or Malignant Mucosal Lesions of the Stomach

**DOI:** 10.3389/fphar.2020.572372

**Published:** 2020-10-02

**Authors:** Bowei Ma, Yucheng Guo, Weian Hu, Fei Yuan, Zhenggang Zhu, Yingyan Yu, Hao Zou

**Affiliations:** ^1^Center for Intelligent Medical Imaging & Health, Research Institute of Tsinghua University in Shenzhen, Shenzhen, China; ^2^Tsimage Medical Technology, Yantian Modern Industry Service Center, Shenzhen, China; ^3^Department of Pathology, Ruijin Hospital, Shanghai Jiao Tong University School of Medicine, Shanghai, China; ^4^Department of General Surgery, Ruijin Hospital, Shanghai Institute of Digestive Surgery, Shanghai Key Lab for Gastric Neoplasms, Shanghai Jiao Tong University School of Medicine, Shanghai, China

**Keywords:** histology, whole-slide imaging, deep learning, convolutional neural network, gastric cancer

## Abstract

Gastric cancer (GC) is one of the leading causes of cancer-related death worldwide. It takes some time from chronic gastritis to develop in GC. Early detection of GC will help patients obtain timely treatment. Understanding disease evolution is crucial for the prevention and treatment of GC. Here, we present a convolutional neural network (CNN)-based system to detect abnormalities in the gastric mucosa. We identified normal mucosa, chronic gastritis, and intestinal-type GC: this is the most common route of gastric carcinogenesis. We integrated digitalizing histopathology of whole-slide images (WSIs), stain normalization, a deep CNN, and a random forest classifier. The staining variability of WSIs was reduced significantly through stain normalization, and saved the cost and time of preparing new slides. Stain normalization improved the effect of the CNN model. The accuracy rate at the patch-level reached 98.4%, and 94.5% for discriminating normal → chronic gastritis → GC. The accuracy rate at the WSIs-level for discriminating normal tissue and cancerous tissue reached 96.0%, which is a state-of-the-art result. Survival analyses indicated that the features extracted from the CNN exerted a significant impact on predicting the survival of cancer patients. Our CNN model disclosed significant potential for adjuvant diagnosis of gastric diseases, especially GC, and usefulness for predicting the prognosis.

## Introduction

Gastric cancer (GC) is one of the leading causes of cancer-related death worldwide, especially in Asia (Van Cutsem et al., 2016; Thrift and El-Serag, 2019). By 2030, deaths from GC globally are predicted to increase from the 15^th^ to the 10^th^ leading cause of cancer related death (Mathers and Loncar, 2006). Due to a lack of effective diagnostic methods, early detection of GC is difficult, which can delay optimal surgical treatment.

Based on histology, GC is divided mainly into “intestinal” and “diffuse” types (Lauren, 1965; Liu et al., 2013). In the former, it is often preceded by several decades of chronic gastritis. The intestinal type of GC is responsible for ~60% of GC cases (Tan and Yeoh, 2015). The stomach is an abdominal organ, so the cancer has often reached an advanced stage or may have metastasized to a distant location by the time significant symptoms appear (Orditura et al., 2014). Therefore, studying the diagnosis and evolution of gastric mucosal lesions is important.

Patients suspected of having GC should undergo endoscopy first. Abnormal tissue seen upon endoscopy will be sent for histology to check for cancerous cells. Histology and pathology reports for biopsies are the “gold standard” in the final diagnosis of cancer worldwide (Thrumurthy et al., 2013). Pathologists visually inspect pathology slides to identify abnormalities, which is a prolonged and tedious process. The human eye is limited in recognizing subtle changes and rare high-dimensional features in tissues, which may result in inter- and intra-observer variability (Raab et al., 2005). Nonstandard subjective judgments can also lead to low diagnostic concordance (Stoler and Schiffman, 2001; Elmore et al., 2015). However, the speed, accuracy and consistency of classification could be improved by application of artificial intelligence (AI) (Gurcan et al., 2009; Ghaznavi et al., 2013).

In recent years, models of “deep learning”, especially “convolutional neural networks” (CNNs) have been shown to perform exceptionally well in computer-vision and pattern-analysis tasks, such as image recognition, semantic segmentation, and object detection (Cruz-Roa et al., 2013; LeCun et al., 2015; Schmidhuber, 2015; Shelhamer et al., 2017; Ren et al., 2017). CNNs can “learn” latent representations of one image to capture complex nonlinear relationships in image data. They can discover more abstract and useful features that make it easier to extract useful information for high-level tasks (Bengio et al., 2013; Wang et al., 2014; Guo et al., 2019).

Research on AI-based cancer histopathology has become an important branch of “digital pathology”. The increased availability of many-gigapixel whole-slide images (WSIs) of tissue specimens has enabled AI to aid detection and classification of cancer (Litjens et al., 2017). Studies have shown that various CNN architectures can be implemented and applied to hematoxylin and eosin (H&E)-stained biopsy slides, such as mitosis detection for biopsy slides of breast tissue and automated detection of basal cell carcinoma (Cruz-Roa et al., 2013; Malon and Cosatto, 2013; Wang et al., 2014). Some scholars have made preliminary achievements in digital-pathology images of GC (Sharma, 2017; Sharma et al., 2017a). A simple CNN architecture for automatic classification of GC using WSIs in histopathology has been described by Sharma et al. (Sharma et al., 2017b), thereby revealing the practicability of AI in digital-pathology research for GC. However, their work has rarely focused on how the deep-learning framework identifies GC lesions, nor how the results might influence the prognosis (Droste et al., 2019; Iizuka et al., 2020).

In the present study, we undertook detection and classification of normal mucosa, chronic gastritis, and intestinal-type GC, which is the most common route of gastric carcinogenesis. This was achieved by proposing a method combining stain normalization, deep CNN, and random forest (RF) classifier. More importantly, we conducted research on how the AI program focused on extracting the morphologic characteristics of gastric mucosal lesions at different stages, which revealed their evolution. Furthermore, we investigated the possible clinical improvement our method could facilitate. We predicted the survival of GC patients by combining the extracted pathologic features from WSIs with clinical follow-up data.

## Materials and Methods

### Dataset and Image Annotation

All gastric-tissue sections were stained with H&E. Then, they were digitalized using a KF-pro-400 scanner (Jiangfeng, Ningbo, China) at 400× magnification. A total of 763 WSIs with manual annotations from the stomach (normal mucosa, chronic gastritis, and GC) were enrolled. Of those, 338 cases were normal gastric tissues (including normal mucosa and smooth muscle), and 118 cases were chronic gastritis. Another 307 cases were intestinal-type GC. All these images were authorized by Ruijin Hospital (Shanghai, China). The study protocol was approved by the ethics review board of Ruijin Hospital. Written informed consent was obtained from patients to use their data.

The digitalized slides were annotated by senior pathologists (YY and FY) with ASAP (an open-source platform for visualizing, annotating and analyzing WSIs; https://computationalpathologygroup.github.io/ASAP/). The key-components of ASAP are: slide input/output, simple image processing, and image viewer. Irregular curves or polygons were used to encircle normal, chronic-gastritis, and GC regions separately in the images. Human-readable Extensible Markup Language (XML) files were generated automatically after manual annotation with a specific format. Data preprocessing involved use of delicate parsing method to extract the annotation information in the XML files to determine the label positions in the digital image.

### Regions of Interest Extraction and Image Segmentation

The size of each WSI can reach 5×10^4^ pixels in both width and height, which is usually beyond the processing power of computers. Hence, we segmented the WSIs into image patches, and then carried out operations on the cut patches. The process of regions of interest (RoI) extraction is shown in **Figure S1**. One canonical method to distinguish the background area from foreground objects is to threshold the image with a “binary mask”. Objects in the WSIs presented various colors and it was inappropriate to use a uniform fixed threshold to distinguish the background and target of all images. Instead, several adaptive threshold methods were applied and compared. The Otsu algorithm (Otsu, 1979) was adopted to determine the threshold of binary-image segmentation by minimizing the intra-class variation (Szegedy et al., 2016a). Then, we undertook a morphologic close operation (which is equivalent to dilation followed by erosion) to close small holes and fill the concave corners in the image. Finally, regions with too small area were abandoned.

In training process, patch-cutting mimics pathologists viewing glass slides from low-power to high-power of a microscope to extract image patches of different sizes: 768×768, 1,024×1,024, 1,495×1,495 and up to 2,048×2,048 pixels. Finally, patches were resized to 299 pixels in weight and height before sending into the CNN. These procedures can train many characteristics at different scales, including the contours of certain lesions and detailed textures. Other too small or too large patches were not adequate for further analyses. The patch size is set as 1,024×1,024 when generating heatmap so that patches can connect to each other by the same side length.

### Stain Normalization and Data Augmentation

To overcome the staining inconsistency of histology slides, multiple researchers have applied operations to standardize specimen colors in histopathological images prior to analysis (Ranefall et al., 1997; Reinhard et al., 2001; Macenko et al., 2009; Tam et al., 2016; Vahadane et al., 2016; Anghel et al., 2019; Ren et al., 2019). One common approach to tackle stain normalization issue is to extract multiple affinities for specific biological substances, and then perform some kind of projection from a preselected reference image to all images. Specifically, color deconvolution methods (Macenko et al., 2009; Vahadane et al., 2016; Anghel et al., 2019) have been utilized extensively in the past decades by transforming the original RGB image into other color space like Lab (Reinhard et al., 2001) and extract the stain vectors. Unsupervised vector estimation methods (Anghel et al., 2019) and generative methods (Ren et al., 2019) have also emerged in the past years. In this study, we applied an internal-feature information of image A to another image B through a specific operation. In brief, a set of characteristic parameters (the RGB color model values of hematoxylin and eosin) are extracted from a reference image, following by a mapping function (Beer-Lamber Law to generate the optical density image and re-assemble the target image’s concentration matrix) that converts the appearance of a given image to the reference image. The parameters are, in general, defined to capture the color distribution of H&E images. As a result, the color distribution of a stain-normalized image will have a great resemblance to the reference image. In general, nuclei are dark-purple (hematoxylin dye) and the cytoplasm is light-pink (eosin dye). To eliminate the influence of the void (white) pixels of the background, we applied a threshold on pixel luminosity to isolate different regions (Macenko et al., 2009). In instances of severe fading, brightness standardization of the images was carried out (Tam et al., 2016). The 2×3 stain matrix, S, was composed of the robust extreme, defined by the two principle eigen-vectors of the optical density (OD) covariance matrix on the angular polar plane. With the extracted stain matrix, the concentration matrix, C, of a given target image could be solved from the equation OD = C × S.

Data augmentation can ease the problem of having few samples. Therefore, we processed affine transformations, such as 30° rotations, migrations by 20% of the dimension, image flipping horizontally and vertically, and shearing by a factor of 0.2. We did not make any extra adjustment on the brightness and contrast of images to preserve the color and texture features of the images after stain normalization.

### Patch Classification at the WSIs-Level and Features Extraction

Due to the limitation of time and hardware, it was impossible to test multiple models on all patches extracted. Therefore, we made a preliminary attempt with a small amount of data on models before using all patches. Models used included Vgg16 (Simonyan and Zisserman, 2014), Resnet50 (He et al., 2016), InceptionResnet v2 (Szegedy et al., 2016a), Densenet169 (Huang et al., 2017) and Inception v3 (Szegedy et al., 2016b). The result of the preliminary attempt showed that Inception v3 was of great potential in this study.

Inception v3 of the open source of Google™ was selected, which contains the module characteristics suitable for pathology tasks (Ker et al., 2019). Inception v3 has been applied in classification tasks in skin cancer (Esteva et al., 2017) and diabetic retinopathy (Gulshan et al., 2016). We added a global average pooling layer, two fully connected layers, and a soft-max layer on the basis of Inception v3. Thus, a modified deep CNN with 43 layers was applied at patch-level classification. The CNN structure is shown in **Figure S2**.

To test the performance of the CNN model for distinguishing different images from various types of gastric diseases, the CNN was trained “from scratch” for 25 epochs with an exponentially decayed learning rate starting at 10^−3^. Then, the set of hyper-parameters with the highest accuracy on the validation set was fine-tuned for another 25 epochs with an exponentially decayed learning rate starting at 10^−4^. In the training process, we used Adam as the optimizer, which has faster convergence speed and can avoid loss function compared with other adaptive learning rate algorithms. The CNN parameters were randomly initialized at the beginning of the first training epoch. Meanwhile, “Cross entropy” was chosen as the loss function corresponding to the soft-max layer.

After obtaining “cancer likelihood maps” from the patches-based classification, we undertook post-processing to extract WSIs-level characteristics. One cancer-likelihood map was created for each WSI, which was an assembled heatmap (H) from enormous patches. One pixel (x, y) in H was generated by assembling the malignant probabilities by taking the highest probability of patches containing the point (*x, y*). That is,

$$H(x,y)=\underset{p\in P}{\max}(I\{(x,y)\in p\})*(\mathrm{Pr}(p!=normal)),$$

where *P* is the set of all patches extracted from the WSI, *p* is one particular patch in the WSI, I(·) is the indicator function, and *Pr*(*p*! = *normal*) is the malignant probability of patch *p*. The tumor-probability threshold (denoted as *P_tumor_*) in the probability section indicates that a pixel in the heatmap is regarded as a tumor pixel if its malignant probability is greater than the threshold.

### RF Classifier for WSIs-Level Classification

To reduce the overfit of the training data due to their randomness, we introduced a RF (Breiman, 2001). A RF is an integrated supervised learning algorithm which ensures that the results of the whole model have high accuracy and considerable generalization performance. The features extracted in the above process are given as the input of the model used for classification at the WSIs-level. The reason that we did not use an end to end approach on WSIs-level classification was that WSIs without preprocessing would cause memory overflow. All the training WSIs were the same as those selected as the training data at the patch-level.

### Visualization of Morphologic Characteristics of Different Gastric Lesions

We wished to construct saliency maps (Simonyan et al., 2013) of the normal mucosa, chronic gastritis, and GC. Hence, we needed to compute the gradients of the unnormalized class score with respect to image pixels, and take the maximum value over red/green/blue (RGB) channels to depict the visually interesting locations in an image. Such topologic representation describes the contribution of each pixel in an image to the confidence of the CNN to classify that image into a specific lesion class. We adopted Grad-CAM (Selvaraju et al., 2020) to produce a coarse localization map highlighting the important regions for predicting the lesions. Grad-CAM takes class-specific gradient information flowing into the penultimate layer of a CNN, and computes an “attention map” showing how intensely the input image activates different channels in the layer with regard to the class. To avoid information loss in the final dense layers, such spatial information in the penultimate layer provides additional guidance. Then, we investigated whether the CNN captured certain cell, nucleus, gland, tissue or stroma features to help identify gastric lesions and make the final decision.

### Survival Analyses

Survival analysis is a crucial ingredient which provides important information about a patient’s prognosis status for treatment design and selection. The combination of clinical features as well as clinicopathological features extracted by machine learning methods like Support Vector Machine (Zhang et al., 2011), Random Forest (Liao et al., 2020), Lasso regression (Li et al., 2019) as well as Deep CNN (Ren et al., 2019) has been proved to substantially enhance the accuracy of survival analysis for different kinds of cancers. To expand the clinical usefulness of our CNN system, we undertook survival analyses which combined the features extracted by the CNN with clinical follow-up data for GC. “Survival” was defined as the percentage of people who survived for a specified period of time. The clinicopathologic features used in RF Model 1 are listed in **Table S2**. We discretized survival duration (right-censored) as <1 year, 1–5 years, and >5 years. Then, the WSIs-level features and clinicopathologic data were fed into a RF classifier. We also compared the prediction performance with the model excluding WSIs-level features for evaluating the effects of WSIs-level features of the CNN system. The Kaplan–Meier estimation method was used (Campos-Filho and Franco, 1988; Miettinen, 2008).

## Results

### Image Patches Produced for CNN Analyses

We used 534 (70%) out of 763 H&E-stained WSIs for the training set, 153 (20%) as the test set, and 76 (10%) as the validation set. The latter was used only to “tune” hyper-parameters in the training process of the CNN. The test set was exposed only when evaluating the performance of the CNN model. The dataset covered >1.6 million image patches in the training set, and 0.4 million image patches in the test set (**Table 1**). In the preliminary attempt, about one fifth of the above patches were used.

**Table 1:** Image numbers at the patch-level of the study.

Images origin	Training set	Test set	Validation set
Normal	544,925	135,446	68,201
Chronic gastritis	544,624	125,783	66,678
Cancer	527,164	138,011	67,452
Total	1,616,713	399,240	202,331

### Stain Normalization and Extraction of Features

Results of the preliminary attempt are listed in **Table S1**. Inception v3 achieved the highest accuracy in the test set among all models, which indicated that the model had great potential in this study. Therefore, Inception v3 was selected as the final CNN structure.

**Figure 1:**
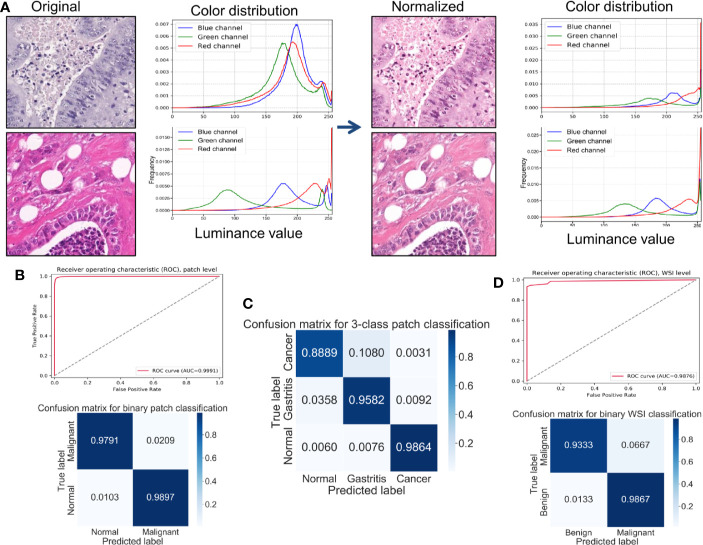
Stain normalization improves the performance of classification at the patch-level and WSIs-level. **(A)** Example of color distributions with or without stain normalization. Left: Two originally stained image patches with their corresponding pixel-color distribution in red/green/blue (RGB) color channels. Right: The same patches after stain normalization and their corresponding pixel-color distribution in RGB color channels. **(B)** Receiver operating characteristic (ROC) curve and normalized confusion matrix for a convolutional neural network (CNN) binary classification model at the patch-level. **(C)** Normalized confusion matrix of a CNN three-class classification model at the patch-level. **(D)** ROC curve and normalized confusion matrix for a random forest binary classification model at the WSIs-level.

All patches were included in the results descried below. In the binary classification (benign *versus* cancer) without stain normalization, the best prediction accuracy on the test set was 98.1%. The prediction accuracy improved to 98.4% after stain normalization. The specificity and sensitivity increased with stain normalization, from 98.2% to 98.9% and from 97.8% to 98.0%, respectively. Stain normalization helped the classification by unifying the distribution of the pixels in the color spaces. **Figure 1A** shows an example of color distribution of the two image patches with and without stain normalization. Despite the original huge color variation of the two patches, the distribution of pixel color was much alike after stain normalization. The improved performance was attributed to the better morphology observed using digital images, so the CNN model could “grasp” directly the different features between them for identification. The receiver operating characteristic curve (ROC) curve for the patch-level classification as well as the corresponding confusion matrix is shown in **Figure 1B**. We further trained and tested the CNN model on the datasets from three classes (normal mucosa, chronic gastritis, and GC), and the best three-class prediction accuracy of the test set was 94.5%. Stain normalization also showed an improvement in three-class patch classification, where the prediction accuracy on the test set with a primary stain was 93.8%. The confusion matrix shown in **Figure 1C** indicates that the reduction in test accuracy (compared with the binary classification) was caused mainly by 10.8% of normal mucosa patches being classified as chronic-gastritis patches.

In everyday clinical practice, whole slices from patients are a combination of normal mucosa, chronic gastritis, and GC. We separated all WSIs into categories: “complete normal WSIs” and “mixture WSIs” with gastritis or GC. After generating cancer-likelihood heatmaps from the patch-level classification, we undertook post-processing to extract WSIs-level characteristics. Referring to relevant study (Wang et al., 2018), forty-four features were extracted from the malignant probability (denoted as *P_tumor_*) heatmap (**Table 2**) in our study, including various morphologic features, such as the long axis length of the largest predicted tumor region. After this feature-extraction process, a RF classifier with 44 extracted features was trained and fine-tuned. The accuracy of the model on the test set was 96.0%, whereas the specificity was 93.3% and sensitivity was 98.7%. The ROC curve for the WSIs-level RF classification as well as the corresponding confusion matrix is shown in **Figure 1D**.

**Table 2:** The 44 features extracted from a heatmap of malignant probability at the whole-slide images (WSIs)-level.

Index	Explanation of feature	Probability remark
1	Total number of tumor regions with an area greater than a threshold	*P_tumor_* ≥0.90, area threshold ≥0.05 total area
2	Area percentage of tumor region over the whole tissue region	*P_tumor_* ≥0.90
3	Area of the largest tumor region	*P_tumor_* ≥0.50
4	Long axis of the largest tumor region	*P_tumor_* ≥0.50
5	Percentage of pixels with a high probability of malignancy	*P_tumor_* ≥0.90
6	Average prediction across the tumor region	*P_tumor_* ≥0.90
7–11	Max, mean, variance, skewness, and kurtosis of the tumor area	*P_tumor_* ≥0.90
12–16	Max, mean, variance, skewness, and kurtosis of the tumor perimeter	*P_tumor_* ≥0.90
17–21	Max, mean, variance, skewness, and kurtosis of tumor compactness (eccentricity)	*P_tumor_* ≥0.90
22-26	Max, mean, variance, skewness, and kurtosis of tumor rectangularity (extent)	*P_tumor_* ≥0.50
27-35	Mean, variance, standard deviation, median, mode, min, max, range, sum of tumor probabilities	n/a
36	Average of malignant probability	n/a
37	Proportion of tumor patches with *P_tumor_ > P_min_*	*P_min_*= 0.999
38	Proportion of tumor patches with *P_max_* ≥ *P_tumor_ > P_min_*	*P_max_* = 0.999, *P_min_* = 0.99
39	Proportion of tumor patches with *P_max_* ≥ *P_tumor_ > P_min_*	*P_max_* = 0.99, *P_min_* **=** 0.95
40	Proportion of tumor patches with *P_max_* ≥*P_tumor_ > P_min_*	*P_max_* = 0.95, *P_min_* **=** 0.9
41	Proportion of tumor patches with *P_max_* ≥ *P_tumor_ > P_min_*	*P_max_* = 0.9, *P_min_* **=** 0.8
42	Proportion of tumor patches with *P_max_* ≥ *P_tumor_ > P_min_*	*P_max_* = 0.8, *P_min_* **=** 0.7
43	Proportion of tumor patches with *P_max_* ≥ *P_tumor_ > P_min_*	*P_max_* = 0.7, *P_min_* **=** 0.6
44	Proportion of tumor patches with *P_max_* ≥ *P_tumor_ > P_min_*	*P_max_* = 0.6, *P_min_* **=** 0.5

### Visualization of Morphologic Characteristics for Different Gastric Lesions

Visualization of morphologic characteristics is a vital function for a deep-learning model because it can show what the model has learnt. We wished to ascertain if the CNN model had seized certain key characteristics of different gastric lesions. Hence, we undertook gradient-weighted class activation mapping (Grad-CAM) and saliency mapping for presentation of patch-level extracted features from the WSIs of different lesions, which corresponded to the evolutionary route of normal mucosa → chronic gastritis → GC. Both visualization styles are presented as heatmaps at patch-level.

Grad-CAM is able to capture certain object contours, which are shown as an overlaid heatmap by blending the computed localization map into the original patch image with 50% transparency. “Warmer” colors correspond to more significant lesions, and *vice versa*. The saliency map was represented by a heatmap indicating the regions whose change would contribute most toward maximizing the predicted probability of that patch belonging to its “true” class (normal, chronic gastritis, or GC). The warmer the color in the heatmap, the higher the possibility the area had to the prediction and *vice versa*.

**Figure 2:**
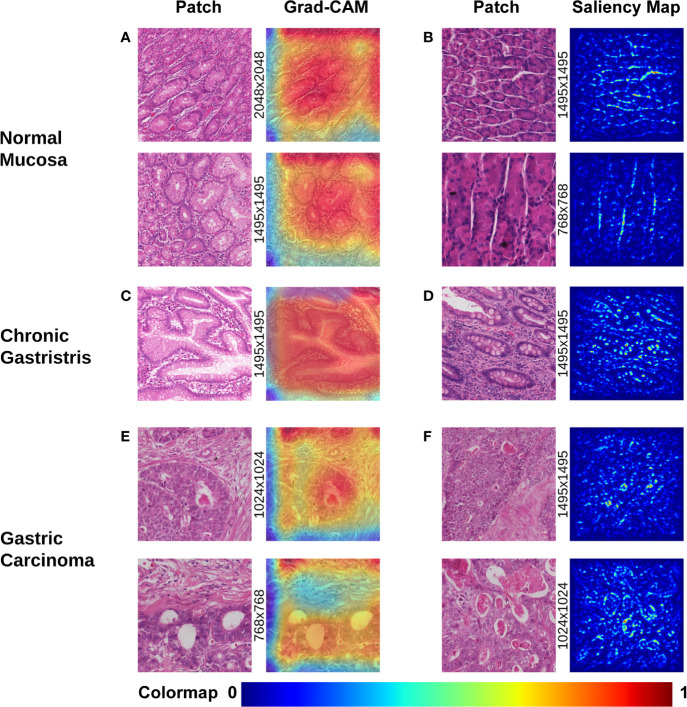
Visualization of different gastric lesions by Grad-CAM and saliency map. **(A)** Normal mucosa. Left: Patches extracted from normal WSIs with 2,048×2,048 pixels and 1,495×1,495 pixels, respectively. Right: Grad-CAM presentation for the patches. **(B)** Normal mucosa. Left: Patches extracted from tubular-gland tissue regions with 1,495×1,495 pixels and 768×768 pixels, respectively. Right: Presentation of the saliency map for the patches. Note that the model captures the contour of the compact glands, which results in lucid lines in the saliency maps. **(C)** Chronic gastritis. Left: Patches extracted from intestinal metaplasia (IM) showing more intracytoplasmic mucin droplets of varying sizes and shapes. Right: Grad-CAM presentation for the patches. **(D)** Chronic gastritis. Left: Patches extracted from an IM lesion with more goblet cells. Right: Presentation of the saliency map for the patches. Note the lucid area of IM is different from normal mucosa. **(E)** Gastric carcinoma. Left: Cancerous patches with more stroma regions. Right: Grad-CAM presentation for the patches. Note that the red area with high attention captures the disordered cancer clusters. **(F)** Gastric carcinoma. Left: Cancerous patches from 1,495×1,495 pixels and 1,024×1,024 pixels, respectively. Right: Presentation of the saliency map for the patches. Note the lucid tubules reveal a significantly irregular shape.

The most prominent characteristics of normal mucosa was the compactness of lining mucosal cells, as well as the morphologic regularity of the structure. Often, morphologic characteristics were seen with patches extracted with a high-power view (patches with pixels of 1,495×1,495 or larger, as shown in **Figure 2A**). Tubular glands were packed closely and separated from each other by the lamina propria. The contour of assembly of the tubular glands with a regular shape was captured clearly, especially in those patches extracted with a low-power view (patches of pixel size 1495×1495 or smaller, as shown in **Figure 2B**).

In chronic gastritis, the significant characteristics were inflammation and intestinal metaplasia (IM), while atrophic lesion of gastric mucosa is the key intermediate stage of transition and development to early GC. IM was captured at high-power magnification at patch-level (patch dimensionality at pixel size 1,024×1,024 or smaller), which disclosed loss of normal mucous glands, as well as the epithelial cells resembling the glands of the intestinal mucosa. The deformed glands could be seen clearly in Grad-CAM presentation (**Figure 2C**) and irregularly shaped intracytoplasmic mucin droplets were observed in the saliency map (**Figure 2D**).

According to Grad-CAM, GC presented with irregularly shaped glands with nuclear pleomorphism and a disordered structure. Distended tubules surrounded by polymorphous cancer cells can be observed at the patch-level in **Figure 2E**. The cancerous features of irregularly shaped and fused neoplastic glands are captured in **Figure 2F**. These glands tended to be fused irregularly or expanded, and filled with inflammatory debris or necrotic cells.

### Features Extracted by CNNs Are Useful for Predicting Outcome

Follow-up data were collected for 273 (88.93%) out of 307 GC patients. The mean duration of follow-up was 46.1 months. The mean age of the study cohort was 61.9 years, and 83.9% of cases were older than 50 years. Clinicopathologic staging was according to the 7^th^ TNM staging criteria of the American Joint Committee on Cancer/International Union Against Cancer Classification for gastric adenocarcinoma (Ahn et al., 2010).

The longest living case was first recorded over 12 years ago. Among them, 118 patients had already died whereas 155 cases were alive. The survival time of patients was discretized into three categories with right-censoring, that is, patients who: (i) died within 12 months; (ii) died within 5 years, but survived for ≥12 months; (iii) survived for ≥5 years. Three RF models with well-tuned hyper-parameters were trained: (i) RF Model 1 (used only clinicopathologic features) (**Table S2**); (ii) RF Model 2 (used 44 extracted features by CNN) (**Table 2**); (iii) RF Model 3 (used all features, including clinicopathologic features and the 44 features extracted by the CNN). With regard to analyses of the RF model, 75% of the data were used for the training set, whereas the remaining 25% were used for the testing set.

**Figure 3:**
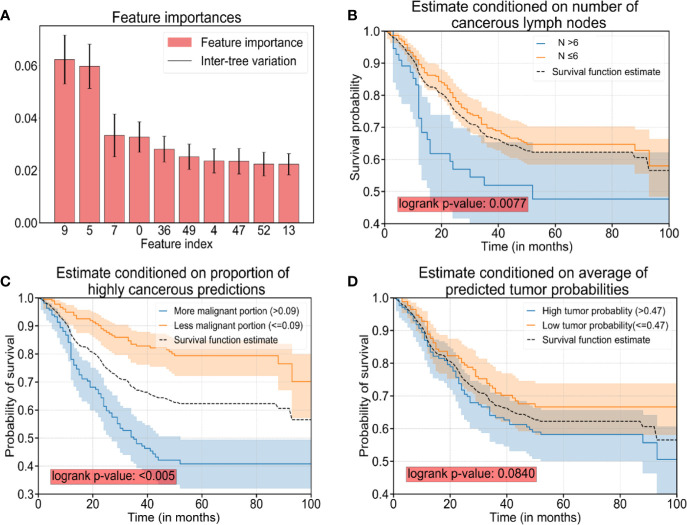
The top-10 significant features and survival analyses. **(A)** The top-10 significant features are presented as bar plots (listed in **Table 3**). **(B)** Survival curves conditioned on observed numbers of cancerous lymph nodes (denoted as N). **(C)** Survival curves conditioned on the proportion of predicted patches with cancerous probability >0.999. **(D)** Survival curves conditioned on predicted tumor probability. Note: The black dotted curves in plot **(B–D)** are the same survival curve estimated for all sample cases as a reference.

After careful tuning of hyper-parameters, the prediction accuracy increased from 92.7% (RF Model 1) to 97.4% (RF Model 3) with the help of the 44 features extracted by the CNN. Hence, a combination of clinicopathologic features with the 44 features extracted by the CNN resulted in an increase in accuracy for predicting survival by 4.7%. In addition, the prediction accuracy was 90.9% with the 44 features extracted by the CNN only (RF Model 2), which suggested that AI-extracted features were important clinically. To clarify the key features of RF Model 3, we inspected the feature importance by computing the number of tree splits one feature determined in the model. Among the top-10 important features (**Table 3**), five of those were clinicopathologic features, whereas the others were features extracted by the CNN (**Figure 3A**). The survival function is presented in **Figure 3B** for index 9 (number of cancerous lymph nodes), **Figure 3C** for index 36 (proportion of prediction probability >0.999) as well as **Figure 3D** for index 49 (average of predicted tumor probability). Index 36 represents the proportion of the patches whose prediction probability is greater than 0.999 in all patches, while index 49 represents the average probability prediction of all patches in each WSI. We segmented the data into two groups according to the feature we were interested in. The median value of the feature value was used for the cutoff. Mathematically, if we were studying the effect of feature *x*, and denoted the median value of *x* in the dataset as $\tilde{x}$, then we compared the survival function of the two segmented groups using the equations (where *Pr*(*X*) is the probability of event *X* and *T* denote the survival time of a patient):

$$S(t)=Pr(T>t|x\geq \tilde{x})\;and\;S(t)=Pr(T>t|x<\tilde{x})$$

**Table 3:** Top-10 features influencing the prognosis.

Index	Name of feature	Importance of feature	Category
9	Number of cancerous lymph nodes	0.0624	clinical
5	Distant metastasis	0.0598	clinical
7	Depth of tumor infiltration	0.0334	clinical
0	Age of patient	0.0328	clinical
36	Proportion of prediction probability >0.999	0.0282	AI
49	Average of predicted tumor probability	0.0253	AI
4	Macroscopic type of tumor	0.0237	clinical
47	Area of the largest tumor region	0.0235	AI
52	Proportion of prediction between 0.5 and 0.6	0.0224	AI
13	Long axis of the largest tumor region	0.0223	AI

The difference in the two survival distributions was tested by the log-rank test. The 95% confidence interval was presented as the transparent shaded area around the curves.

## Discussion

A deep CNN model for aiding the digital-pathology diagnosis of gastric lesions was designed and implemented. Beyond the classification of histopathologic images, our CNN model also captured features behind the CNN procedure to a certain extent. Currently, CNNs are applied not only in digital pathology, but also in computed tomography (CT) scans, ophthalmoscope images, and ultrasound images (Esteva et al., 2017; De Fauw et al., 2018; Coudray et al., 2018; Philbrick et al., 2018; Falk et al., 2019; Li et al., 2019). Studies (Macenko et al., 2009; Iizuka et al., 2020) have revealed that AI can identify various lesions with a level of competence observed by imaging experts.

Unlike CT scans or ultrasound images, which are grayscale images, WSIs of tissues are color images and more likely to suffer from color variations due to different staining conditions. Staining inconsistencies may be attributed to multiple factors: dye, staining protocols of laboratories, fading, and digital scanners (Vahadane et al., 2016; Roy et al., 2018). To ease the adverse impact upon the analytic accuracy of AI, multiple scholars have tried various methods to standardize color distribution in images (Khan et al., 2014; Vicory et al., 2015; Bejnordi et al., 2016; Samsi et al., 2018). However, use of a single transformation function for each channel is rarely sufficient. The method of “intensity centering” and histogram equalization enables automatic extraction of reference-stain vectors by finding the fringe of pixel distributions in the optical-density space, but yields poor estimation of the stain vectors in the presence of strong staining variations (Tam et al., 2016). In contrast to the diverse colors of natural images, pathologic images often have a standard staining protocol. The color of pathologic images is affected severely by dyes, storage times, and fading. To tackle such problems, the luminosity of histology slides must be considered because dust and microbes will dim the transparent background and deteriorate the efficacy of stain normalization. We proposed a method involving integration of a brightness-standardization process into stain normalization to filter-out the influence of different levels of brightness and luminosity of the slides.

In analyses of WSIs, considering the gigantic magnitude, Sharma and colleagues designed a program to extract small patches at a fixed scale (e.g., 256×256 pixels) and trained a deep CNN to classify these small patches (Sharma et al., 2017a). One possible shortcoming of their method is that there are often some spatial correlations between neighboring patches, and discarding such information may result in unstable prediction results.

Here, we proposed a method to remedy such loss in spatial correlation. We combined patches at different scales into the training process: 2,048×2,048, 1,495×1,495, 1,024×1,024, and 768×768 pixels. In this way, not only was the spatial correlation preserved but also the textual contours of different lesions at different scales were captured.

Previously, analyses of the interpretability of pathologic slides of gastric tissue relied merely on simple probability heatmaps (Sharma et al., 2017a). The generated probability loses information about the internal process in deep-learning models.

We applied complex visualization methods onto gastric lesions to visualize activation in the deep-learning model and the logic behind its decision. Multiple approaches, including Grad-CAM, saliency maps, and variations in saliency-map computations, such as rectified saliency (Zeiler and Fergus, 2014) and guided saliency (Springenberg et al., 2014), were tested. By examining saliency-map and Grad-CAM visualizations, we showed some morphological features in the figure, which might be of significance in the process of recognition of pathological images by CNN model. Moreover, by carefully inspecting the morphologic features captured by deep-learning models, it is possible to identify various diseases. To uncover the mystery of the CNN model in analyses of medical images, we outlined the key characteristics underlying AI processing, and extracted 44 features, which had roles for discriminating a normal mucosa, chronic gastritis, and intestinal-type GC. Some of those features could be interpreted based on pathologic morphology, others from computing language. More importantly, the features extracted by the CNN were not only useful for classifying different gastric lesions, they also had a role in predicting the prognosis. This is the first time that crucial features have been revealed for prognostic diagnosis by a CNN model. In the present study, >88% of GC cases were followed up clinically for a long time. Hence, we assessed the possible influencing factors for clinical outcomes. We found that that certain features extracted by AI played an important part in assessing disease severity and predicting the prognosis for patients with GC.

Our study had one main limitation. About 30–40% of GCs are classified as diffuse or rare types, which our current system could not identify. The diagnosis of those types of GC, is part of our research which will be carried out in the future and more relevant WSI images have already been prepared. The CNN model we constructed may have a greater role in AI-assisted differential diagnoses for diffuse or rare types of GC in the future. Besides, we will collect cases of different stages between normal type and GC in the next study for better survival analysis.

## Conclusions

**Figure 4:**
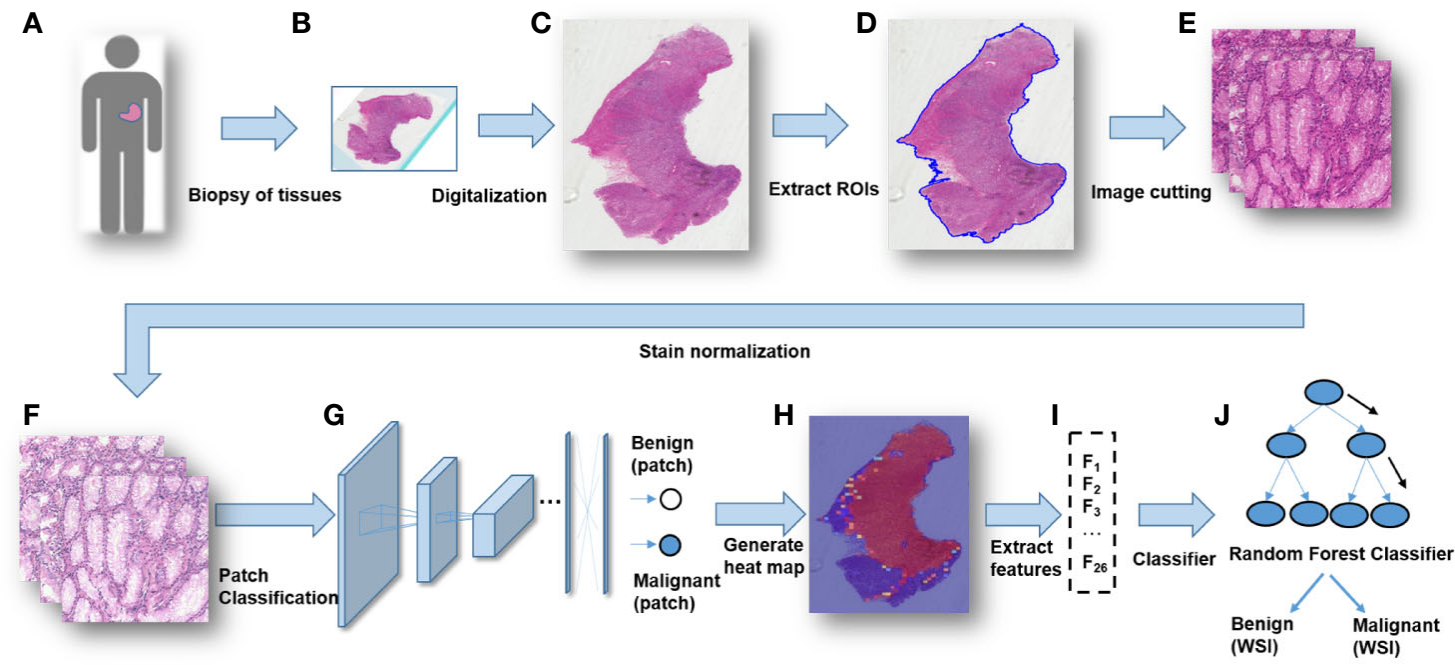
The workflow of classifying gastric lesions by a convolutional neural network (CNN) model. **(A)** Patients undergo curative surgery or biopsy. **(B)** Preparation of histopathologic slides. **(C)** Digitalization of the glass slides into WSIs. **(D)** RoI extraction for WSIs. **(E)** Cutting patches from WSIs with multiple scales. **(F)** Stain normalization for the patches. **(G)** Patches-level classification through a deep CNN model. **(H)** Generation of a malignancy probability heatmap with patch-level classification results. **(I)** Extraction of WSIs-level features from the malignancy heatmap. **(J)** Whole-slide images (WSIs)-level classification for patients.

A modified Inception v3 CNN was applied to classify gastric diseases. We segmented WSIs into patches on various scales, and normalized the patches stain. We obtained a good performance for discriminating normal mucosa, chronic gastritis, and intestinal-type GC based on 44 key features at the WSIs-level. The heatmap of malignant probability could provide guidance for pathologists to rapidly notice suspicious regions at the WSIs-level (**Figure 4**). More importantly, certain features extracted from the CNN model revealed clinical importance for predicting disease severity and the prognosis. The future direction of GC study could integrate clinicopathologic features, extracted AI features, as well as genomic features to guide “precision medicine”.

## Data Availability Statement

All datasets presented in this study are included in the article/**Supplementary Material**.

## Ethics Statement

This study was approved by the institutional review board of Ruijin hospital, and a written informed consent was obtained from the participants of this study.

## Author Contributions

BM, YG, and WH were responsible for the design, implementation and experiments on data preprocessing, data augmentation, training/prediction pipeline, visualization analyses and survival analyses. FY and ZZ collected and assessed clinical information, and YY and HZ supported that work. All authors contributed to the article and approved the submitted version.

## Funding

This project was supported by the Shanghai Science and Technology Committee (18411953100), National Key R&D Program of China (2017YFC0908300, 2016YFC1303200, 2018YFF0301102 and 2018YFF0301105), National Natural Science Foundation of China (81772505), Cross-Institute Research Fund of Shanghai Jiao Tong University (YG2017ZD01), Innovation Foundation of Translational Medicine of Shanghai Jiao Tong University School of Medicine (15ZH4001, TM201617 and TM 201702), and Technology Transfer Project of Science & Technology Department of Shanghai Jiao Tong University School of Medicine.

## Conflict of Interest

The authors declare that the research was conducted in the absence of any commercial or financial relationships that could be construed as a potential conflict of interest.
