# Supplementary material for: Artificial Intelligence-Based Multiclass Classification of Benign or Malignant Mucosal Lesions of the Stomach
Source: Front Pharmacol. 2020 Oct 2;11:572372. doi: 10.3389/fphar.2020.572372 (PMC7562716; doi:10.3389/fphar.2020.572372)
Supplement: Supplementary file 1 [file DataSheet_1.docx]

Supplementary Material

## Supplementary Figure

##
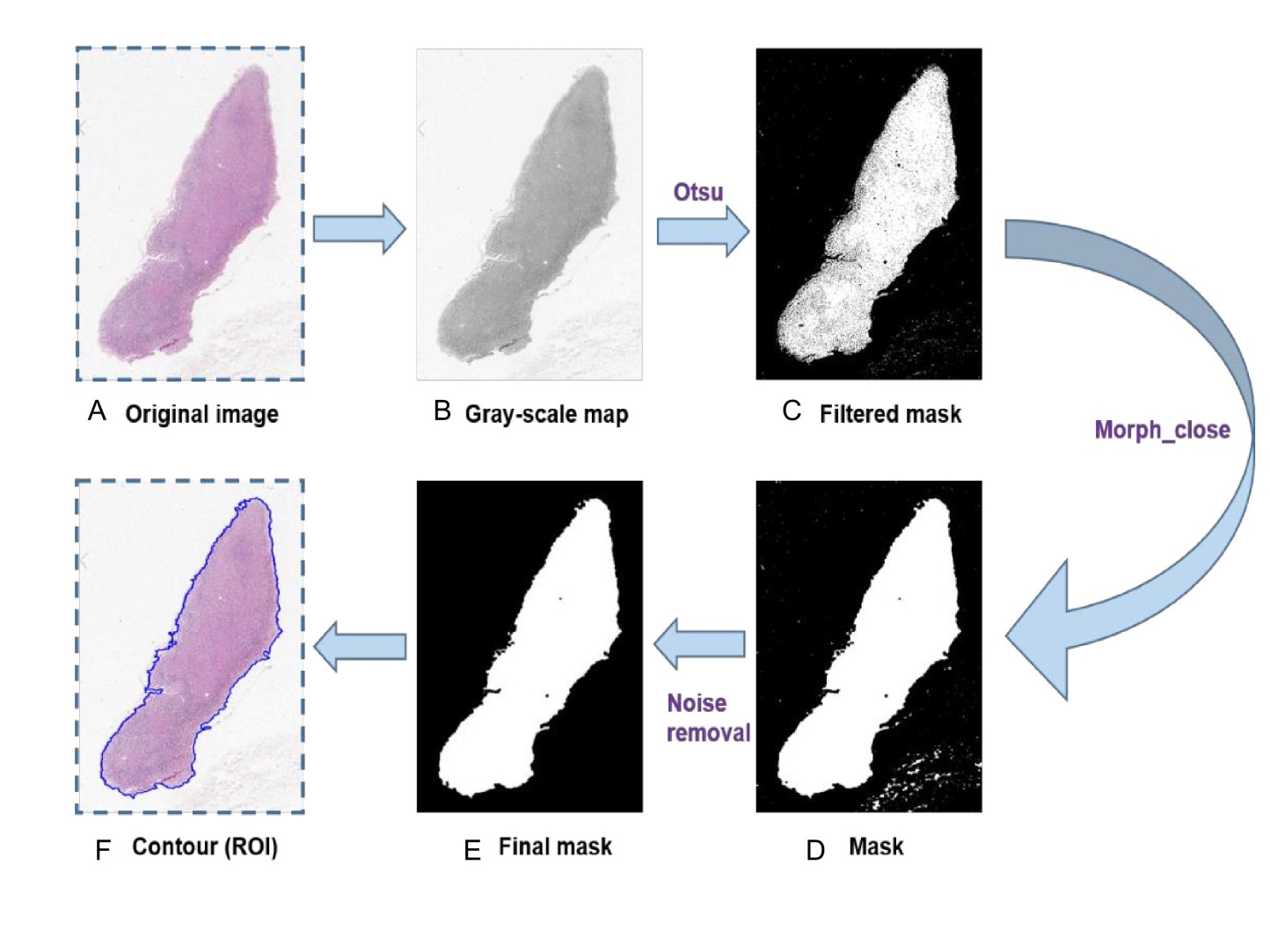


**Supplementary Figure 1.** Process of RoI extraction. (A) Original image. (B) Grayscale “mask” of the original image. (C) Mask obtained after application of the Otsu threshold method. (D) Mask obtained after application of “morphologic closure”, which can remove noisy spots in the result of the previous process. (E) Final mask: independent small black regions have been abandoned. (F) Result of R0I extraction: patches will be extracted in the region surrounded by a blue line.

##
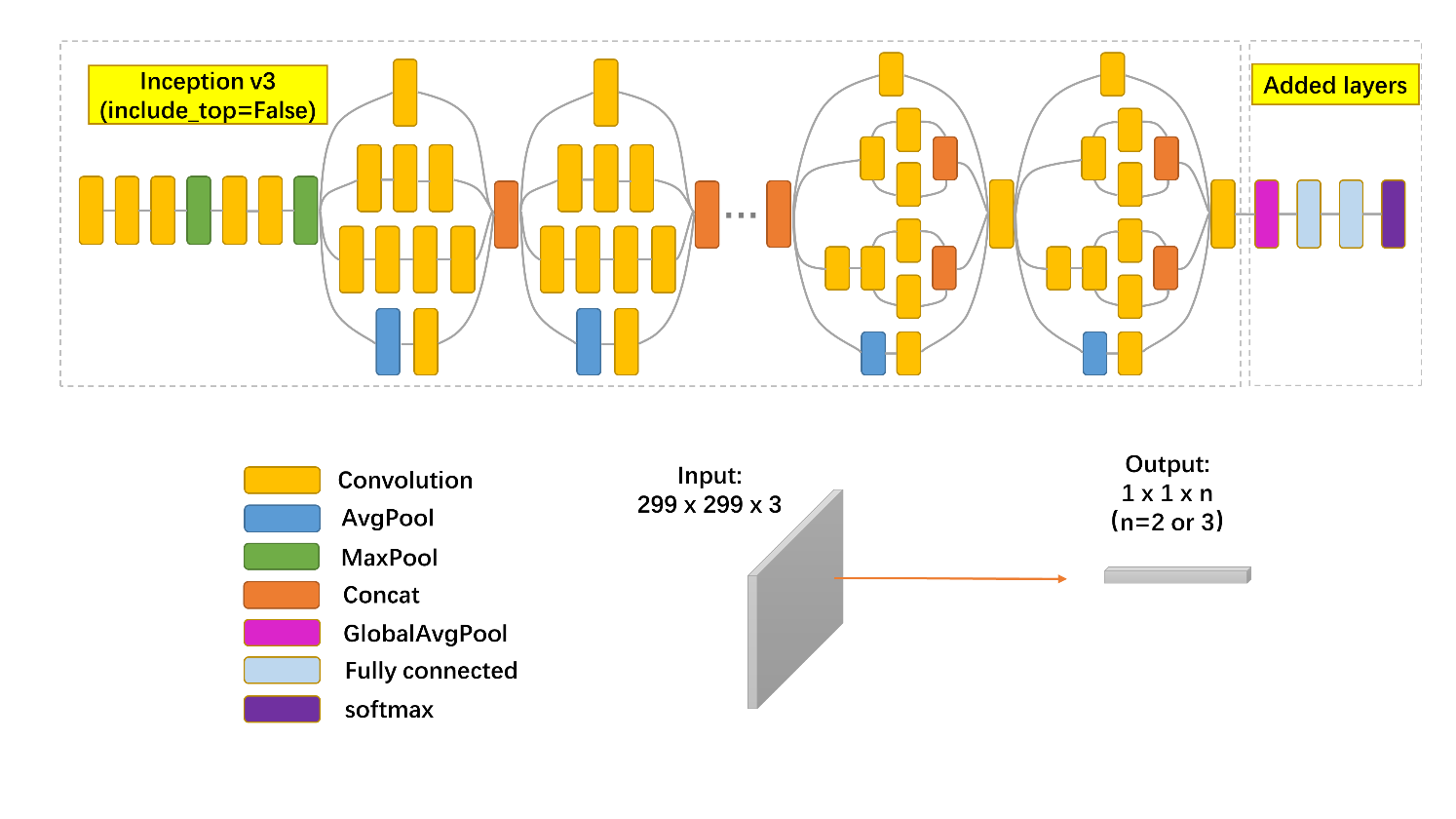


**Supplementary Figure 2.** Structure of the modified Inception v3 CNN. Different modules in the network are represented by different colors. A global average pooling layer, two fully connected layers and a soft-max layer are added after the inception main architecture. Input size is 299×299×3, while the output size is 1×1×n (n is set as 2 or 3 according to number of categories).

## Supplementary Table

**Supplementary Table 1.** Results of preliminary experiment based on the part of patches

| Model | Accuracy of valid set | Accuracy of test set |
| --- | --- | --- |
| Vgg16 | 84.99% | 89.70% |
| InceptionResnet v2 | 82.24% | 87.72% |
| Densenet169 | 76.67% | 77.98% |
| Resnet50 | 82.86% | 89.26% |
| Inception v3 | 82.87% | 90.06% |

**Supplementary Table 2.** Clinicopathologic features used in RF Model 1

| Index | | Feature | Remarks |
| --- | --- | --- | --- |
| 1 | Sex of the patient | | n/a |
| 2 | Age of the patient | | In years |
| 3 | Location of tumor region | | Categorized for simplicity |
| 4 | General type of disease | | n/a |
| 5 | Length of the long-axis of the tumor | | In centimeters |
| 6 | Node stage in the TNM staging system | | n/a |
| 7 | Number of metastatic lymph nodes | | n/a |
| 8 | Depth of tumor infiltration | | T in the TNM system |
| 9 | Indicator for metastasis | | Binary indicator, M in the TNM system |
| 10 | Indicator for vessel invasion | | Binary indicator |
